# Diversity-enhanced reconstruction as plug-in defenders against adversarial perturbations

**DOI:** 10.3389/frai.2025.1665106

**Published:** 2025-09-25

**Authors:** Zeshan Pang, Xuehu Yan, Shasha Guo, Yuliang Lu

**Affiliations:** ^1^College of Electronic Engineering, National University of Defense Technology, Hefei, Anhui, China; ^2^Anhui Province Key Laboratory of Cyberspace Security Situation Awareness and Evaluation, Hefei, Anhui, China

**Keywords:** adversarial attack, adversarial defense, diversity training, computer vision, deep learning

## Abstract

Deep learning models are susceptible to adversarial examples. In large-scale deployed services, plug-in defenders efficiently defend against such attacks. Plug-in defenders take two approaches to mitigate adversarial effects: input reconstruction and random transformations. Existing plug-in defense lacks diversity in transformation formulation due to the inherent feature preservation nature, which leads to vulnerability under adaptive attacks. To address this issue, we propose a novel plug-in defense named Diversity-enhanced Reconstruction (DeR). DeR counters adversarial attacks by frequency-aware reconstructors with enhanced diversity. Specifically, we design the reconstructors as a U-Net backbone with additional frequency components. The reconstructors are trained on the proposed DeR loss, which optimizes the reconstruction and diversity objectives jointly. Once trained, DeR can produce heterogeneous gradients and be applied as a plug-in defense. We conduct extensive experiments on three datasets and four classifier architectures under strict adversarial settings. The results demonstrate the superior robustness of DeR compared to state-of-the-art plug-in defense and the efficiency of DeR in real-time processing.

## 1 Introduction

While deep learning models are adopted as core parts of various intelligent systems, they face the threat of adversarial attacks. By adding minor perturbations to the input images, adversaries can mislead the models without being noticed by human inspectors. The misconduct of deep learning systems may trigger system failure and cause severe user harm. In autonomous driving, a patch printed with adversarial perturbations on the road sign is enough to make the intelligent system mistake it as another sign, causing the vehicle to violate the traffic rules or even leading to crashes (Suryanto et al., 2023).

The security concerns of deep learning models have drawn the focus of researchers. Some approaches, such as adversarial training (Zhang et al., 2019; Wang et al., 2023b; Ho et al., 2022) and ensemble learning (Kariyappa and Qureshi, 2019; Yang et al., 2020, 2021; Chen et al., 2024), enhance the model robustness during training. However, retraining deployed models may interrupt the services and introduce considerable computational and time overhead. Moreover, the evolution of attack techniques puts continuous demand on developing the robustness of models. Thus, plug-in defenders that mitigate adversarial effects without modifying pre-trained models are more plausible for large-scale deployed intelligent systems.

Plug-in defenders purify inputs before passing them to downstream models, aiming to remove adversarial perturbations while preserving benign features. Two major approaches for achieving this goal are input reconstruction and random transformations. Input reconstruction (Liao et al., 2018; Wang et al., 2023a; Huang et al., 2024; Yang et al., 2019; Hill et al., 2021; Yoon et al., 2021; Nie et al., 2022) utilizes the reconstruction error to eliminate adversarial perturbations. Random transformations (Raff et al., 2019; Pérez et al., 2021; Chen et al., 2022; Wang et al., 2024) disable adversarial perturbations by common image processing such as rotation and denoising. However, both approaches are susceptible to adaptive attacks (Athalye et al., 2018a,b; Lee and Kim, 2023).

**Figure 1:**
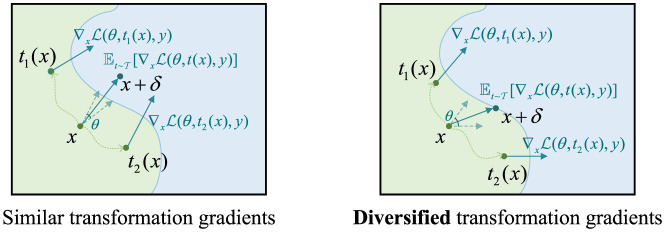
Difference between similar transformation and diversified transformation when computing the expectation of gradients. The expectation of gradients of diversified transformation deviates from the fastest direction of approximating the decision boundary.

Because the attack path always exists in a deterministic inference, static plug-in defenders are easily breached by calculating the gradients to cover both the defender and the classifier. In the meantime, randomization introduced by the defense module can be reduced to an ensemble of multiple deterministic inferences with a chosen distribution at test time. Assume there exist two primary defensive transformations *t*_1_ and *t*_2_, then the attack paths for the two transformations are $\nabla_{x}L\left(\theta ,t_{i}\left(x\right),y\right),i=1,2$. An adaptive attack utilizing the EOT technique reduces the attack path to ${\text{E}}_{t\in T}\left[\nabla_{x}L\left(\theta ,t\left(x\right),y\right)\right]$. When the transformations share a similar attack path in the feature space, the attack effectively induces the sample across the decision boundary, as illustrated in the left diagram of Figure 1. If the transformations' attack paths are diversified, as in the right diagram of Figure 1, the adversarial attacks are deviated, less effective. A larger perturbation strength will be needed to fool the defended classifier.

Out of such intuition, we propose Diversity-enhanced Reconstruction (DeR) as a plug-in defense. DeR is composed of two reconstructors that simultaneously reconstruct the input samples. The reconstruction process introduces diversified gradients and thwarts adversarial attacks. DeR's reconstructors are equipped with frequency-aware components, making them capable of perceiving minor features and producing more diversity.

DeR is efficient as a plug-in defense against adversarial attacks. First, the diversified reconstructors effectively thwart the search for gradient-based adversarial examples. Meanwhile, the frequency components provide sufficient gradient diversity, averting retraining downstream classifiers, and preserving accuracy on clean inputs. In addition, the plug-in characteristic characterizes DeR as flexible in accommodating multiple classifier architectures without extra training. The experiments conducted on CIFAR-10, SVHN, and Tiny-ImageNet with four classifier architectures (ResNet He et al., 2016, VGG Simonyan and Zisserman, 2014, Wide ResNet Zagoruyko and Komodakis, 2016, ConvMixer Trockman and Kolter, 2023) verify our method's robustness promotion and efficiency.

The main contributions of our study are as follows:

- We propose DeR as a novel plug-in defense against adversarial attacks. The reconstructors in DeR thwart adversarial attack by producing diversified gradients.
- We introduce frequency-aware U-Net as our backbone of reconstructors. The frequency components enhance the reconstructors' sensitivity toward minor distribution shifts, which provides redundancy for producing diversity.
- The performance of DeR is validated on three real-world datasets and four classifier architectures. The results demonstrate our method's superiority under adversarial attacks both in robustness and efficiency. Under PGD and AutoAttack, DeR brings up the classifier accuracy from lower than 10% to over 20% and 30%.

The remainder of this study is organized as follows. In Section 2, we will state the problem in adversarial defense and introduce related works in plug-in defense. Then in Section 3, the formulation of our method is elaborated. The experimental results and analysis are demonstrated in Section 4. Finally, in Section 5, we will summarize the stduy.

## 2 Preliminaries and related works

This study focuses on defending against adversarial attacks, which significantly threaten deployed intelligent systems. Many works have delved into the security problem under such attacks. From the perspective of deployment convenience and adversarial robustness, plug-in defense is a common practice. However, adversarial examples generated with full knowledge of the defenders pose a critical threat to existing plug-in defenses. In Section 2.1, we will first formulate the threats of adversarial examples. After that, we will briefly review the progress in plug-in defense and analyze its vulnerability under strong adversaries.

### 2.1 Adversarial examples

Szegedy (2013) first identified deep learning models' vulnerability to minor perturbations. Adversarial attacks craft adversarial examples, which are similar to natural samples but can mislead the classifier, by imposing imperceptible perturbations to natural inputs. When given a classifier *f* with weights θ, the optimization of adversarial perturbation δ based on sample *x* is conducted as


(1) $$\begin{array}{l} \delta =\delta +\alpha \cdot \text{sign}\left(\nabla_{x}L\left(\theta ,x,y\right)\right), \end{array}$$

where α denotes the step size of each iteration, and L(·) is the loss function depending on the task of the target model, such as cross-entropy loss for classification models. The search can be performed in one step (Goodfellow et al., 2015) or iteratively (Madry et al., 2018), depending on the adversaries' computational budget and attack strength. The perturbations are bounded within a given radius ϵ to ensure invisibility.

Plug-in defense complicates the gradients computed by the adversaries through defensive pre-processing. When the exact parameters of the defense model are unknown, adversaries still implement attacks according to Equation 1, which is referred to as gray-box attacks. Otherwise if the pre-processing *t*(·) is also exposed, one can implement white-box attacks according to Equation 2,


(2) $$\begin{array}{l} \delta =\delta +\alpha \cdot \text{sign}\left(\nabla_{x}L\left(\theta ,t\left(x\right),y\right)\right). \end{array}$$

However, back-propagating the transformation *t*(·) may be intractable. In which cases, gray-box attacks may be more efficient than white-box ones (Athalye et al., 2018a) due to the feature-preserving nature of the transformation. Furthermore, BPDA (Athalye et al., 2018a) technique can bypass the defense by approximating the transformation as an identical mapping. As for defense that obfuscate gradients by randomization, the EOT (Athalye et al., 2018b) technique is often effective in estimating optimal gradient direction under a given distribution of transformations. By utilizing these attack techniques, the adversaries can design more effective adversarial examples in a white-box scenario. Thus, the white-box scenario of adversarial defense is considered more rigorous. This study mainly considers white-box attacks to evaluate our defense method more reliably.

### 2.2 Plug-in defense

The intuition behind plug-in defense is pre-processing the inputs to deliberately disrupt adversarial perturbations while maintaining the benign features. There are mainly two approaches to eliminate adversarial effects, including input reconstruction and randomized augmentation.

Early works (Meng and Chen, 2017; Liao et al., 2018; Samangouei et al., 2018; Yoon et al., 2021) utilize the loss of details in image reconstruction to eliminate adversarial perturbations, which can be modeled as a denoising process. Since the reconstruction process is fixed, the cascade of denoiser and classifier is degraded to a deterministic target model, which white-box attacks can easily breach. Later, randomization is introduced to the reconstruction process (Yang et al., 2019; Dai et al., 2020; Hill et al., 2021; Nie et al., 2022). Stochastic elements such as noise obfuscate the search for optimal perturbations. However, to ensure the accuracy of downstream classifiers, the reconstruction outputs are designated to approximate the original inputs, that is, *t*(*x*)≈*x*. Thus, the gradient in Equation 2 can be approximated as


(3) $$\begin{array}{l} \nabla_{x}L\left(\theta ,t\left(x\right),y\right)=\frac{\partial L\left(\theta ,t\left(x\right),y\right)}{\partial t\left(x\right)}\cdot \frac{\partial t\left(x\right)}{\partial x}\\ \;\approx \frac{\partial L\left(\theta ,x,y\right)}{\partial x}\cdot \;1, \end{array}$$

where 1 is the all-ones tensor of the same shape as the inputs produced by back propagating the identical mapping, *c* is the number of channels, and *m* and *n* represent the input image's shape. Equation 3 implies a shortcut for adversarial perturbations searching that evades obfuscated gradients and is the main idea of BPDA technique.

Other works (Raff et al., 2019; Pérez et al., 2021; Chen et al., 2022) pave an alternative path to reduce the efficacy of adversarial perturbations by combining conventional image augmentation as a defensive transformation. The transformation typically includes rotation, scaling, shifting, and blurring. Generally, assume the number of possible transformations is *N* and they form a transformation set $T=\left\{t_{1},t_{2},...,t_{N}\right\}$. In inference, the inputs are processed by *m* transformations randomly or strategically selected from T and the outputs of models are integrated for final prediction $F\left(T_{s},x\right)=\underset{t_{i}\in T_{s}}{\sum }f\left(t_{i}\left(x\right)\right)$, where $T_{s}\subset T$. Then, gradients in Equation 2 can be approximated by EOT technique,


(4) $$\begin{array}{l} {\text{E}}_{T_{s}\subset T}\left[\nabla_{x}L\left(\theta ,T_{s}\left(x\right),y\right)\right]={\text{E}}_{t\in T}\left[\nabla_{x}L\left(\theta ,t\left(x\right),y\right)\right]. \end{array}$$

While this approach benefits from simple processing steps, combining random transformations provides little diversity in gradients and thus gains limited robustness.

Recently, Li et al. (2023) combine the above two approaches with a transformation network that learns the optimal affine transformation to offset adversarial effects. The transformation network, however, is still differentiable and deterministic, making it vulnerable to white-box attacks.

To summarize, plug-in defense faces the trade-off between input fidelity and transformation diversity. One widely adopted approach is input denoising, which is vulnerable to BPDA attacks. The other approach randomly transforms the inputs susceptible to EOT attacks. The main reason for both approaches' failure is that the diversity of transformations is limited, such that the approximated or estimated gradients remain aligned with the proper adversarial direction toward decision boundaries.

## 3 Methodology

**Figure 2:**
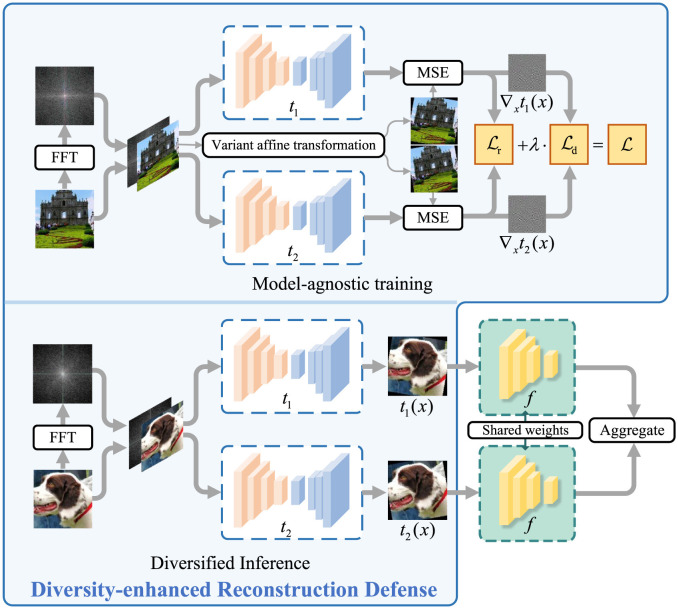
Training and inference procedure of DeR (Images reproduced from: Learning Multiple Layers of Features from Tiny Images, Krizhevsky, 2009).

To address the above problem, we propose DeR. As illustrated in Figure 2, DeR defends adversarial attacks with multiple reconstructors that apply diversified transformations to the inputs. The reconstructors adopt a simplified U-Net structure with frequency processing units. The network design is detailed in Section 3.1. To balance the trade-off between adversarial robustness and input fidelity, we propose the DeR loss that enables the reconstructors to learn the transformations while producing diversified gradients, which will be formulated in Section 3.2. The training of reconstructors is model-agnostic, and the trained networks can be transferred to homogeneous classifiers. In the inference step, the classifier takes the reconstructed images as inputs, respectively, and integrates the outputs.

### 3.1 Frequency-aware U-net

**Figure 3:**
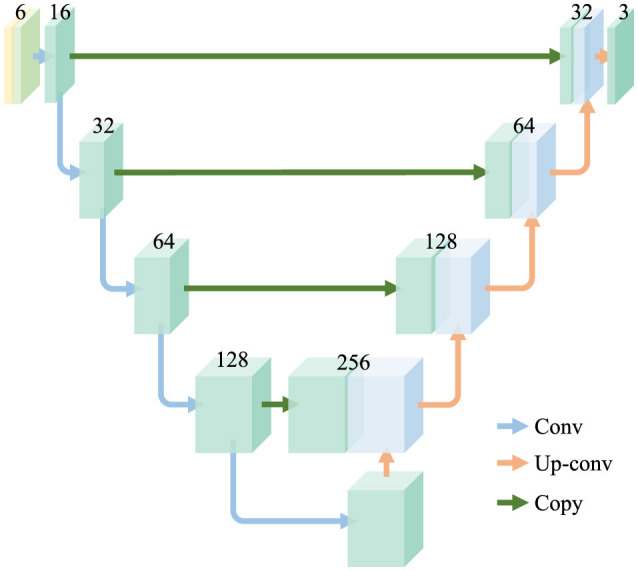
Architecture of the reconstructors. The frequency and spatial images are jointly forwarded to the networks. There are mainly two feature extraction blocks in the reconstructors, which are convolution layer (Conv) and Up convolution layer (Up-conv). The features extracted are copied and concatenated to form skip connections.

U-Net (Ronneberger et al., 2015) was first proposed for semantic segmentation and is also widely used in image reconstruction tasks. We adopt a simplified U-Net as the backbone of reconstructors, as shown in Figure 3. The original U-Net structure is reduced to five layers for computational efficiency. The shape of features in the hidden layers is identical to preserve redundancy for diversity. The outermost layer receives the concatenation of the input and its spectrum produced by Fast Fourier Transformation (FFT), which results in the outermost layer having six input channels. The frequency components enable the network to capture the subtle features in the frequency domain and further provide redundancy for diversity.

### 3.2 Diversity training

Diversity training is the key for reconstructors to produce homogeneous gradients while preserving image features. To provide more redundancy for gradient diversity, DeR is composed of two reconstructors. This is also the most computationally efficient setting for generating necessary defense deviations. Without loss of generality, assume the input *x* is processed by the paired independent reconstructors in $T=\left\{t_{1},t_{2}\right\}$, respectively, the integrated prediction of classifier *f* is $F\left(T,x\right)=\frac{1}{2}\overset{2}{\underset{i=1}{\sum }}f\left(t_{i}\left(x\right)\right)$. As analyzed in Section 2, enhancing gradient diversity is essential for adversarial robustness. We will first declare the measurement of gradient diversity in our scheme, and then, we will elaborate the training objective and loss function for DeR.

#### 3.2.1 Gradient diversity

For starters, we will analyze the components of gradients in plug-in defense. The gradient of outputs w.r.t. *x* writes


(5) $$\begin{array}{l} \nabla_{x}F\left(T,x\right)=\frac{1}{2}\overset{2}{\underset{i=1}{\sum }}\frac{\partial f\left(t_{i}\left(x\right)\right)}{\partial x}. \end{array}$$

Assume the reconstructed inputs still belong to original manifold and $\frac{\partial f\left(t_{i}\left(x\right)\right)}{\partial t_{i}\left(x\right)}\approx \frac{\partial f\left(x\right)}{\partial x}$, with the chain rule, Equation 5 can be rewritten as


(6) $$\begin{array}{l} \nabla_{x}F\left(T,x\right)=\frac{1}{2}\overset{2}{\underset{i=1}{\sum }}\frac{\partial f\left(t_{i}\left(x\right)\right)}{\partial t_{i}\left(x\right)}\cdot \frac{\partial t_{i}\left(x\right)}{\partial x}\approx \frac{\partial f\left(x\right)}{\partial x}\cdot \frac{1}{2}\overset{2}{\underset{i=1}{\sum }}\frac{\partial t_{i}\left(x\right)}{\partial x}. \end{array}$$

According to Equation 6, we can obfuscate the gradients by diversifying the gradients produced by reconstructors without modifying the classifiers. An aggressive way of obfuscating the gradients is forcing $\overset{2}{\underset{i=1}{\sum }}\frac{\partial t_{i}\left(x\right)}{\partial x}$ to approximate zero, which means the gradients of reconstructors offset each other. However, this profoundly hinders the optimization of reconstruction. Alternatively, we take the cosine similarity to measure the gradients' diversity following (Kariyappa and Qureshi, 2019). Cosine similarity measures the deviation of vectors in Euclidean space, which accords with the diversity intuition illustrated in Section 1. The cosine similarity of gradients produced by any two reconstructors is defined as


(7) $$\begin{array}{l} CS\left(\nabla_{x}t_{i}\left(x\right),\nabla_{x}t_{j}\left(x\right)\right)=\frac{<\nabla_{x}t_{i}\left(x\right),\nabla_{x}t_{j}\left(x\right)>}{\left|\nabla_{x}t_{i}\left(x\right)|\cdot |\nabla_{x}t_{j}\left(x\right)\right|}. \end{array}$$

Enlarging the expectation of Equation 7 on input samples is the diversity objective in DeR.

#### 3.2.2 DeR loss

DeR loss is comprised of reconstruction loss and diversity loss. The reconstruction loss is based on the reconstruction objective, which is designated as


(8) $$\begin{array}{l} \underset{\phi_{i}}{\min}{\text{E}}_{x\sim D}\left(∥t_{i}^{\prime }\left(x\right)-t_{i}\left(x\right){∥}_{2}\right), \end{array}$$

where $t_{i}^{\prime }\left(\cdot \right)$ is an affine transformation that the *i*th reconstructor fits. For all the reconstructors, the corresponding $t_{i}^{\prime }$ differs from each other to provide redundancy for gradients diversity. The reconstruction loss writes


(9) $$\begin{array}{l} L_{\text{r}}=\overset{2}{\underset{i=1}{\sum }}∥t_{i}\left(x\right)-t_{i}^{\prime }\left(x\right){∥}_{2}. \end{array}$$

According to Equation 4, variant transformations do not necessarily lead to diversified gradients. Here, we introduce the affine transformations $T^{\prime }=\left\{t_{i}^{\prime }\left(\cdot \right),i=1,2\right\}$ as auxiliary reconstructors. The diversity loss for DeR is formulated as


(10) $$\begin{array}{l} L_{\text{d}}=\log(\sum_{1\leq i<j\leq 2}\exp(CS(\nabla_{x}t_{i}(x),\nabla_{x}t_{j}(x)))\\ \;+\sum_{1\leq i,j\leq 2}\exp(CS(\nabla_{x}t_{i}(x),\nabla_{x}t_{j}^{\prime }(x)))), \end{array}$$

where we adopt Log-Sum-Exp (LSE) to optimize Equation 7 for convergence stability. In Equation 10, the first term constrains the diversity among reconstructors, and the second term diversifies the reconstructors' gradients from the affine transformations. Equation 10 deviates the reconstructors' gradients and limits the effectiveness of perturbations generated by approximating the process as affine transformations.

By combining reconstruction loss and transformation diversity loss, the joint loss function of training DeR writes


(11) $$\begin{array}{l} L_{\text{DeR}}=L_{\text{r}}+\lambda \cdot L_{\text{d}}, \end{array}$$

where λ is the balancing coefficient that controls the trade-off between input fidelity and transformation diversity. Enlarging λ forces the reconstructors to diversify more with each other but may sacrifice the input fidelity. The quantitative analysis of λ's effect on adversarial robustness and input fidelity will be demonstrated in Section 4.4.

## 4 Experiments

This section presents the experiment results that validate the effectiveness of DeR. The experimental setup is detailed in Section 4.1 in five aspects. Section 4.2 compares the robustness of DeR and other baseline methods to verify the effectiveness of DeR as a plug-in defense. Then in Section 4.3, we demonstrate the efficiency of DeR in inference time. Finally, Section 4.4 analyzes the impact of major components in the DeR scheme.

### 4.1 Experimental setup

#### 4.1.1 Datasets

We validate the proposed method on CIFAR-10 (Krizhevsky, 2009), SVHN (Netzer et al., 2011), and Tiny-ImageNet (Le and Yang, 2015), which are widely adopted real-world datasets. The CIFAR-10 dataset consists of 60,000 images with size 32 × 32 from 10 classes of daily items. SVHN dataset is the dataset for digits recognition in street view and also contains 10 classes. Images in SVHN are split to training set of 73,257 samples and test set of 26,032 samples, with size of 32 × 32. Tiny-ImageNet is a subset of ImageNet (Deng et al., 2009) consisting of 100,000 images of 200 classes (500 for each class) downsized to 64 × 64 colored images.

#### 4.1.2 Classifiers

The experiments are conducted on four widely used classifier architectures: ResNet, WideResNet, VGG, and ConvMixer. The ResNet classifiers are the 32-layer version for the CIFAR-10 dataset described in the original paper (He et al., 2016) and are abbreviated as ResNet-32. The WideResNet (Zagoruyko and Komodakis, 2016) classifiers' parameters are *depth* = 28 and *k* = 10, abbreviated as WRN-28-10. The VGG classifiers adopted are the same as the 11-layer model in the paper of Simonyan and Zisserman (2014), abbreviated as VGG-11. ConvMixer (Trockman and Kolter, 2023; Yoshioka, 2024) classifiers are implemented based on ConvMixerTiny training codes in the repository.^1^

The base classifiers are trained for 100 epochs with the initial learning rate of 0.001, decaying to 0.0001 at the 60th epoch. Random rotation and horizontal flip are applied to the training samples as data augmentation. For baseline plug-in methods and DeR, the classifiers are base classifiers.

#### 4.1.3 Baseline methods

The baseline methods include plug-in and ensemble defense. The plug-in methods adopted from existing works are HGD (Liao et al., 2018), EBMDefense (Hill et al., 2021), and DefenseTransformer (Li et al., 2023), which cover preprocessing approaches including denoising, randomization, and deep-learning-based transformation. HGD denoises the inputs with high-level representation guidance. EBMDefense utilizes randomization to obfuscate gradients. DefenseTransformer trains a transformation network that imposes optimal affine transformations to the inputs. HGD models are trained on FGSM with ϵ = 0.03. EBMDefense models adopt the same configurations as the official codes.^2^ DefenseTransformer models are trained on PGD with ϵ = 0.03. In addition, we compare DeR with random affine transformations to elaborate that the robustness of DeR is not attributed to the affine mapping and that DeR gains robustness from diversity-trained reconstructors. The adopted transformations are the same as those that the DeR reconstructors learn.

We compare DeR with ensemble methods because DeR involves the inference and integration of multiple outputs. In addition, diversity enhancement is also the focus for ensemble methods, and our gradient diversity regularizer derives from ensemble defense. The ensemble methods are based on the Gradient Alignment Loss (GAL) (Kariyappa and Qureshi, 2019) and the Enhancing Adversarial Robustness through Diversity that Supports Robustness (EADSR) (Chen et al., 2024) method. GAL is the first method that observes and utilizes the correlation between robustness and gradient similarity, closely related to our work. EADSR further enhances the diversity of ensembles by differentiated predictions. All the ensemble methods are trained and tested with three sub-models. The balance coefficient in GAL is 0.5, following the same setting in the paper. The parameters of EADSR follow the same configurations as in the paper. All the baseline methods are aligned in clean accuracy for fair comparison.

#### 4.1.4 Adversarial configurations

The algorithms used to generate adversarial examples are FGSM (Goodfellow et al., 2015), Autoattack (Croce and Hein, 2020), PGD (Madry et al., 2018), and SMER (Tang et al., 2024). FGSM, PGD, Autoattack, and SMER perturbations are constrained within the *L*_∞_ norm 8/255. PGD attack is iterated for 30 steps with a step size of 2/255.

We consider the white-box scenario where the adversaries are assumed to have full knowledge of the defense methods and the classifiers' weights. Whitebox attacks are more reliable in evaluating the effectiveness of defense methods since the adversaries may take advantage of all the information about the defender. Since some methods may gain robustness by obfuscated gradients, the attacks are implemented in an adaptive way that may ignore the gradients of the pre-processing steps. We report the worst case of robustness for all the methods for fair comparison. The worst-case attack configurations are listed in Table 1. The attacks following Equation 2 without adaptive attack techniques are denoted by Whitebox. Meanwhile, in the BPDA setting, the gradients are approximated by differentiable mappings.

**Table 1:** Worst-case attack settings for different defense methods.

**Method**	**Attack settings**
HGD	Whitebox
EBMDefense	BPDA
DefenseTransformer	Whitebox
GAL	Whitebox
EADSR	Whitebox
Affine transformations	EOT
DeR (ours)	BPDA + EOT

#### 4.1.5 Implementation details

The reconstructors adopt the simplified U-Net architecture with frequency components detailed in Section 3.1. We use two reconstructors to produce diversified gradients jointly trained by the DeR loss in Equation 11. The training is conducted on three datasets and iterated for 40 epochs over the training set. The learning rate was initially set to 0.01 and decreased by a factor of 10 in the 20th epoch. The parameter λ is set to 1.5 unless stated otherwise.

### 4.2 Adversarial robustness

We evaluate the robustness of our and baseline methods on three datasets, taking the worst-case settings in Table 1. Specifically, in EBMDefense, the differentiable mapping is an identical mapping. In DeR, the differentiable mappings are corresponding affine transformations. BPDA can be applied alongside EOT, which estimates the expectation of gradients of transformed inputs. We only implement the SMER attack on ensemble defense. EBMDefense is partially incompatible with AutoAttack, so we omit its evaluation. In addition, the assessment of ConvMixer is only implemented for plug-in defense due to the limitations of computing power. The robustness of defense is measured by the accuracy of downstream classifiers on adversarial examples. We also list the classifiers' accuracy on clean samples to compare the input fidelity of different defenders.

#### 4.2.1 Robustness under gradient-based attacks

DeR thwarts the search for adversarial perturbations through diversified gradients. Based on the analysis in Section 3, the scattered gradients decrease the effectiveness of gradient-based attacks. We verify these insights by evaluating the robustness of our methods and the most recent works on plug-in defense on FGSM, PGD, and AutoAttack. The results on CIFAR-10, SVHN, and Tiny-ImageNet are shown in Tables 2–4, respectively. For Tiny-ImageNet, we did not experiment with the ConvMixer classifier due to device limitations. The U-Net structure of DeR is altered, the kernel size and stride of Convolutional and TransposeConvolutional layers are changed to 4 and 2 for computational efficiency and perceptual ability, and the hidden dimension is changed to 128. Similarly, the stride of the last down-sampling layer of DefenseTransformer's U-Net is changed to 2.

**Table 2:** Classifier accuracy (%) with DeR and plug-in defense under gradient-based attacks on CIFAR-10.

**Classifier**	**Method**	**Attack**			
		**None**	**FGSM**	**PGD**	**AutoAttack**
ResNet-32	HGD	89.2	33.1	0	0
	EBMDefense	88.8	20.1	1.0	–
	DefenseTransformer	84.0	11.3	0	0
	Affine transformations	89.2	25.5	0	4.8
	DeR (ours)	86.1	**38.3**	**19.5**	**28.2**
VGG-11	HGD	87.5	35.4	0	0
	EBMDefense	89.8	12.8	1.3	–
	DefenseTransformer	84.0	2.8	0	0
	Affine transformations	86.0	19.0	0.3	6.4
	DeR (ours)	86.2	**37.7**	**20.1**	**30.8**
WRN-28-10	HGD	85.3	23.5	0	0
	EBMDefense	88.5	11.1	0.3	–
	DefenseTransformer	77.0	7.6	0	0
	Affine transformations	88.4	20.6	0.3	3.9
	DeR (ours)	88.7	**38.6**	**22.3**	**29.2**
ConvMixer	HGD	91.1	**64.6**	0	0
	EBMDefense	90.6	17.3	3.6	–
	DefenseTransformer	83.5	26.1	0	0
	Affine transformations	90.4	14.0	0	1.2
	DeR (ours)	88.5	44.0	**35.9**	**44.8**

The bold font represents the highest accuracy, and the underlined data correspond to the second highest performance.

**Table 3:** Classifier accuracy (%) with DeR and plug-in defense under gradient-based attacks on SVHN.

**Classifier**	**Method**	**Attack**			
		**None**	**FGSM**	**PGD**	**AutoAttack**
ResNet-32	HGD	95.4	**92.3**	0.2	0.7
	EBMDefense	95.4	32.7	2.5	–
	DefenseTransformer	89.2	9.6	0	0
	Affine transformations	95.2	38.8	3.3	12.5
	DeR (ours)	94.9	46.5	**22.8**	**30.7**
VGG-11	HGD	93.3	**72.4**	1.4	0.7
	EBMDefense	94.0	34.7	5.3	–
	DefenseTransformer	88.6	12.1	0	0
	Affine transformations	94.7	38.3	4.2	15.0
	DeR (ours)	94.5	52.8	**27.8**	**35.8**
WRN-28-10	HGD	95.2	**79.1**	0.2	0.4
	EBMDefense	96.2	38.5	5.0	–
	DefenseTransformer	88.5	14.6	0	0
	Affine transformations	94.8	40.7	6.6	11.7
	DeR (ours)	96.1	48.4	**24.1**	**31.5**
ConvMixer	HGD	95.5	**93.9**	1.2	2.2
	EBMDefense	96.1	45.9	6.2	-
	DefenseTransformer	91.5	52.4	0	0
	Affine transformations	95.1	55.0	8.2	16.1
	DeR (ours)	95.6	57.4	**27.1**	**34.4**

The bold font represents the highest accuracy, and the underlined data correspond to the second highest performance.

**Table 4:** Classifier accuracy (%) with DeR and plug-in defense under gradient-based attacks on Tiny-ImageNet.

**Classifier**	**Method**	**Attack**			
		**None**	**FGSM**	**PGD**	**AutoAttack**
ResNet-32	HGD	47.6	5.7	0	0
	EBMDefense	40.9	2.7	**4.8**	–
	DefenseTransformer	26.0	0.4	0	0
	Affine transformations	47.7	3.0	0	2.8
	DeR (ours)	46.7	**10.4**	0.9	**2.9**
VGG-11	HGD	49.3	**20.9**	0	0
	EBMDefense	47.7	6.3	4.6	–
	DefenseTransformer	40.8	5.4	0	0
	Affine transformations	53.2	7.3	0	2.8
	DeR (ours)	47.8	20.3	**9.1**	**5.7**
WRN-28-10	HGD	60.2	20.5	0	0
	EBMDefense	53.9	6.4	7.3	–
	DefenseTransformer	49.5	3.3	0	0
	Affine transformations	59.8	12.9	0.2	2.4
	DeR (ours)	54.4	**24.1**	**12.4**	**5.5**

The bold font represents the highest accuracy, and the underlined data correspond to the second highest performance.

On CIFAR-10 dataset, DeR achieves the highest accuracy under all the attacks except for FGSM attacks on the ConvMixer classifier. Especially under PGD and AutoAttack, DeR exceeds the baseline accuracy of lower than 0.05 with a promotion beyond 20%–40%. The robustness of HGD is not substantial since it does not generalize to multiple attack settings. On SVHN and Tiny-ImageNet datasets, DeR surpasses all the other baselines under PGD and AutoAttack except for EBMDefense on ResNet-32. Although HGD attains the best accuracy under the FGSM attack, it fails to defend iterative attacks, with the accuracy below 0.1. Our method demonstrates comprehensive resistance to all the tested attacks.

By comparing DeR with other plug-in defenses, we can conclude that DeR demonstrates better robustness under gradient-based attacks in most cases. The results indicate that DeR benefits from diversified transformations and degrades the effectiveness of adversarial examples.

#### 4.2.2 Robustness under transferable attack

Since our methods involve the combination of multiple networks, transferable attacks designed for ensemble defense can be applied to evaluate DeR's robustness. We compare DeR with ensemble defense to further demonstrate the superiority of our method under transferable attacks. The baseline methods are GAL (Kariyappa and Qureshi, 2019) and EADSR (Chen et al., 2024). In GAL, the authors also discussed the generation of diversified sub-models by diversity training. EADSR is a SOTA ensemble approach. We evaluate the robustness of DeR and ensemble methods under SMER (Tang et al., 2024) attack. SMER utilizes the diversity in the ensemble to promote the transferability of adversarial examples. It is based on the FGSM iteration, which makes it highly transferable among parallel defense models. The attack settings are listed in Table 1.

The classifier accuracy with DeR and baseline ensemble defense under SMER attacks is shown in Table 5. In most cases, DeR surpasses the baseline ensemble methods with significant improvement. In other instances, DeR achieves comparable accuracy. The results indicate that the diversified gradients in DeR effectively thwart transferable attacks.

**Table 5:** Classifier accuracy (%) with DeR and baseline ensemble defense under SMER attacks.

**Datasets**	**Classifier**	**Method**		
		**GAL**	**EADSR**	**DeR (ours)**
CIFAR-10	ResNet-32	5.5	0.9	**9.0**
	VGG-11	1.2	1.1	**14.1**
	WRN-28-10	7.7	10.2	**14.7**
SVHN	ResNet-32	2.0	**17.8**	15.8
	VGG-11	0.8	12.4	**20.4**
	WRN-28-10	0	**22.9**	19.7

The bold font represents the highest accuracy.

### 4.3 Inference efficiency

**Figure 4:**
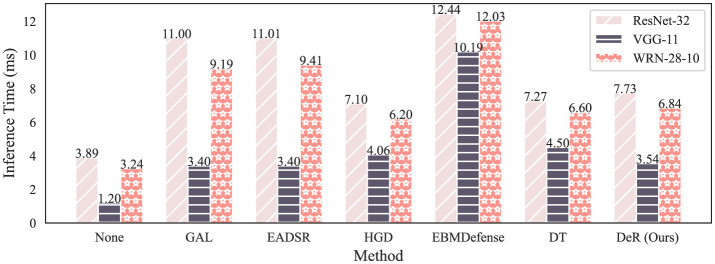
Inference time of different defense methods tested on CIFAR-10.

DeR is also more efficient in inference as a plug-in defense. Figure 4 demonstrates the inference time of different defense methods. The results are tested on CIFAR-10 dataset with an RTX 3050 OEM GPU and an Intel Core i7-13700. The inference time is tested by averaging over 1,000 random samples to obtain reliable results. In test time, the batch size is set to 1. It can be seen from the plot that DeR takes less time in inference compared to ensemble defense and is faster or comparable to baseline plug-in defense. The results indicate that DeR is efficient in inference and can reduce the time delay in real-time services while defending against adversarial attacks.

Table 6 demonstrates the throughput of DeR and baseline methods with batch sizes 1, 16, and 128. We test the throughput for 100 batches for each batch size and average the results. With batch size 1, DeR's throughput is slightly lower than that of HGD, but when the batch size increases, DeR's processing speed climbs steeply, and with batch size 128, DeR is the fastest among all the defense methods. The results indicate that DeR is suitable for real-time image processing.

**Table 6:** Throughput (image/sec) comparison of different defense methods on CIFAR-10.

**Classifier**	**Batch**	**Defense method**						
	**Size**	**None**	**GAL**	**EADSR**	**HGD**	**EBM**	**DT**	**DeR (ours)**
ResNet-32	1	203	64	60	**97**	42	68	88
	16	4,222	1,007	1,176	**2,088**	890	1,663	2,048
	128	42,944	11,964	13,702	17,820	10,588	17,446	**18,903**
VGG-11	1	792	282	**286**	180	100	208	261
	16	13,065	3932	**4,578**	2,584	1,526	3,494	3,794
	128	108,147	34,155	**40,425**	30,406	14,059	28,692	37,512
WRN-28-10	1	301	78	76	**145**	77	109	136
	16	4,448	1,392	1,298	2,188	1,314	2,118	**2,421**
	128	38,814	14,838	14,238	22,269	11,932	19,467	**23,176**

The bold font represents the highest accuracy, and the underlined data correspond to the second highest performance.

We further test the memory usage of different defense methods. The results are shown in Table 7. The batch sizes are 1, 16, and 128. We tested the memory usage on 100 batches for every batch size and averaged the results. On ResNet-32, DeR uses slightly more memory than ensemble methods. This is because ResNet-32 is a small model, and the memory usage of defense methods is determined by the volume of the sub-models and the ensemble size. At the same time, DeR reconstructors have sizes comparable to those of the classifier. However, DeR is still the best plug-in method. On the other two models, the memory usage gains in DeR are little. This is the benefit of DeR's model-agnostic training and light-weighted reconstructors. From the data provided in Table 7, we validate DeR's efficiency in memory usage.

**Table 7:** Memory usage (MB) comparison of different defense methods on CIFAR-10.

**Classifier**	**Batch**	**Defense method**						
	**Size**	**None**	**GAL**	**EADSR**	**HGD**	**EBM**	**DT**	**DeR (ours)**
ResNet-32	1	9.9	13.7	13.6	53.3	**13.2**	54.4	14.5
	16	10.0	**13.7**	**13.7**	52.3	23.5	54.6	14.8
	128	13.6	17.5	**15.0**	78.0	89.9	77.2	26.1
VGG-11	1	43.4	115.3	115.3	86.4	**46.0**	87.4	47.9
	16	44.3	116.5	116.5	88.5	**46.6**	87.5	48.1
	128	44.6	117.9	117.4	98.7	76.7	102.5	**64.4**
WRN-28-10	1	149.4	428.6	428.6	191.4	**150.5**	191.9	153.1
	16	150.1	427.2	427.0	193.5	163.6	194.0	**154.8**
	128	155.2	440.3	431.3	208.1	256.1	214.3	**173.3**

The bold font represents the highest accuracy, and the underlined data correspond to the second highest performance.

The above results show DeR's efficiency in time delay, throughput, and memory usage, making it an efficient plug-in defense.

### 4.4 Ablation study

In this subsection, we examine the effects of major components in DeR. The first aspect concerns the design of the DeR loss term, including the LSE process of diversity loss, the diversity enhancement induced by DeR loss, and the impact of the hyper-coefficient λ on DeR loss. The second aspect inspects the design of reconstructors, including frequency component efficacy of reconstructors, DeR defense with more reconstructors, and DeR defense with different reconstructor structures.

#### 4.4.1 LSE in DeR loss

**Figure 5:**
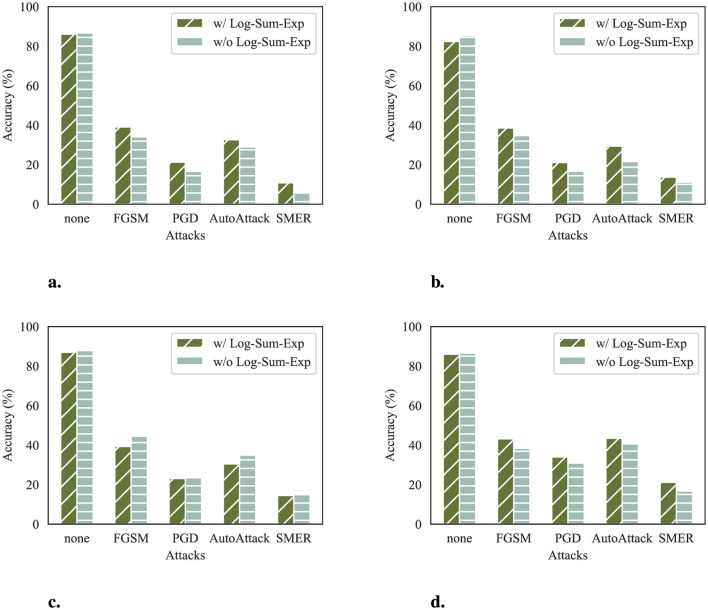
Classifier accuracy (%) of reconstructors with and without LSE. **(a)** Accuracy on ResNet-32. **(b)** Accuracy on VGG-11. **(c)** Accuracy on WRN-28-10. **(d)** Accuracy on ConvMixer.

In DeR loss, we utilize the LSE for the summation of gradient similarity. The effect of this smoothness is to enhance the reconstructors' diversity optimization process, which is reflected in the robustness of defense results. To demonstrate the differences between reconstructors with and without LSE, we train reconstructors with a non-smoothed version of DeR loss and compare their robustness with the original design. For fair comparison, the non-smoothed reconstructors are trained with α = 0.1 to align with the original reconstructors on clean accuracy. The attack results are demonstrated in Figure 5.

The results show that reconstructors trained with LSE are more robust to adversarial attacks at the same level of clean accuracy on most classifiers. This implies that LSE helps the reconstructors to capture and retain image features, which is preferred in the subsequent recognition stage of classifiers. Thus, using LSE in DeR loss makes our method suitable for more classifier architectures.

#### 4.4.2 Enhanced diversity

**Figure 6:**
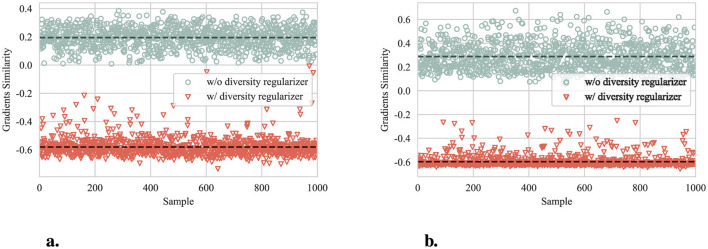
Gradient similarity of reconstructors with and without diversity regularizer. The dashed lines represent the corresponding average. **(a)** Gradient similarity on CIFAR-10. **(b)** Gradient similarity on SVHN.

Figure 6 shows the gradient similarity of reconstructors with and without diversity regularizer. The results are obtained by calculating the LSE of the gradients' cosine similarity produced by reconstructors on 1,000 random samples from the test set of CIFAR-10 and SVHN datasets. In both datasets, the reconstructors trained with diversity regularizer exhibit significantly lower gradient similarity, with an averaged reduction of approximately 0.8. The results verify the proposed diversity constraint.

#### 4.4.3 Impact of different λ

**Figure 7:**
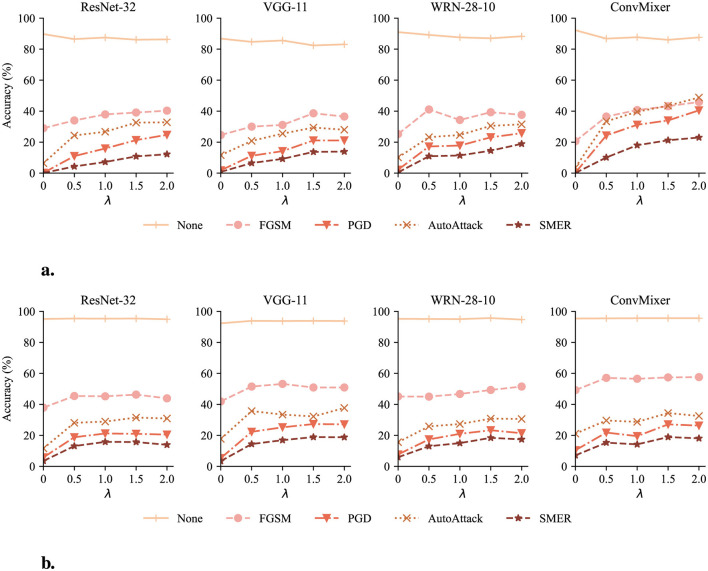
Classifier accuracy with DeR defense of λ ranging from 0 to 2. The None attack represents the accuracy on clean samples. **(a)** Classifier accuracy on CIFAR-10. **(b)** Classifier accuracy on SVHN.

As elaborated in Section 3.2.2, the hyperparameter λ controls the balance between accuracy and robustness. We investigate the influence of λ by testing with reconstructors trained on different values of λ. Figure 7 demonstrates the impact of λ on classifier accuracy.

In most cases, as λ increases, the accuracy under adversarial attacks continuously increases and reaches a plateau or slightly drops after λ = 1.5. Meanwhile, the classifier accuracy on clean samples (lines of the None attack) suffers slight degradation. However, in models with sufficient parameter redundancy, such as WRN-28-10 and ConvMixer, DeR shows potential in higher robustness with larger λ. The results confirm the relationship between λ and the accuracy-robustness trade-off. Increasing λ brings stronger resistance to adversarial attacks but sacrifices the classifier performance on clean samples. In addition, this trend also indicates that the loss term controlled by λ, i.e., the gradient diversity loss, plays a significant role in adversarial robustness.

#### 4.4.4 Impact of frequency components

DeR introduces frequency components to the reconstructors for better convergence and performance on diversity learning. In this section, we investigate the impact of frequency components to verify our design. To this end, we train reconstructors without frequency components for comparison. The compared reconstructors take the same architecture as in Section 3.1 except that the input layer takes only spatial information as input and has three channels. All the reconstructors are trained with the settings described in Section 4.1.

**Figure 8:**
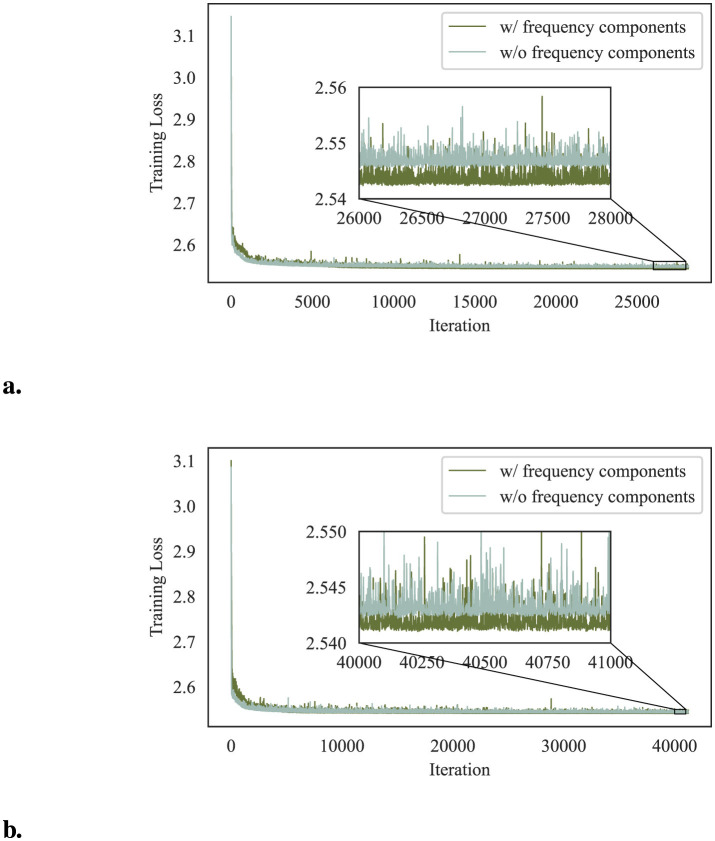
Training losses of reconstructors with and without frequency components. **(a)** Training losses on CIFAR-10. **(b)** Training losses on SVHN.

Figure 8 demonstrates the training losses of reconstructors with and without frequency components. The losses of the last several iterations are zoomed in for comparison. From the perspective of training losses, reconstructors with frequency components converge to a lower value, indicating better performance in balancing reconstruction quality and gradient diversity.

**Figure 9:**
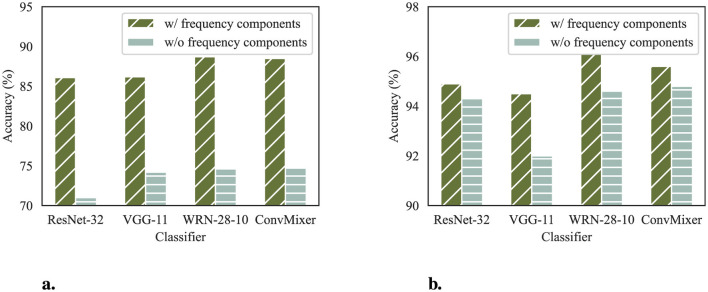
Clean accuracy of classifier defended by reconstructors with and without frequency components. **(a)** Classifier accuracy on CIFAR-10. **(b)** Classifier accuracy on SVHN.

We also compare the accuracy of both architectures on clean samples. The results are shown in Figure 9. The classifier accuracy on clean samples corroborates the observation of the differences in training losses. Reconstructors without frequency components fail to balance conflicts of benign feature preservation and adversarial perturbations removal, resulting in poorer classification performance on CIFAR-10 and SVHN datasets.

From the above analysis, we can conclude that the frequency components in DeR are essential for the reconstructors to produce redundancy and generate diversified gradients while preserving input fidelity.

#### 4.4.5 DeR with more reconstructors

Although we experiment only on DeR with two reconstructors, the DeR loss is compatible with scenarios where the number of reconstructors *N*>2. We experimentally trained three reconstructors to fit three affine transformations, respectively. Two transformations are fixed to rotation –10 and 10 degrees (rotation ±10°). For the third transformation, we tried out three settings, which are rotation –5 degrees (rotation −5°), shifting along the *x*-axis for 0.15 of the image width (shifting 0.15), and cropping the central 0.9 part of the original image and resizing to the original shape (scaling 0.9). For comparison, we list the best results of baseline methods for each classifier in the first row. All the reconstructor groups are trained with α = 1.5. The experimental results on CIFAR-10 are provided in Table 8.

**Table 8:** Classifier accuracy (%) with different DeR reconstructor combinations on CIFAR-10.

**Classifier**	**Affine transformations**	**Attack**			
		**None**	**FGSM**	**PGD**	**AutoAttack**
ResNet-32	Baseline best	89.2	33.1	1.0	4.8
	Rotation ±10°	86.1	**38.3**	**19.5**	**28.2**
	Rotation ±10°, rotation −5°	85.1	35.2	8.9	1.7
	Rotation ±10°, shifting 0.15	86.8	36.8	11.7	21.3
	Rotation ±10°, scaling 0.9	86.0	37.7	8.4	15.6
VGG-11	Baseline best	89.8	35.4	1.3	6.4
	Rotation ±10°	86.2	**37.7**	**20.1**	**30.8**
	Rotation ±10°, rotation −5°	85.3	34.0	13.6	19.6
	Rotation ±10°, shifting 0.15	86.2	32.3	15.1	20.9
	Rotation ±10°, scaling 0.9	87.5	33.0	10.1	18.4
WRN-28-10	Baseline best	88.5	28.1	2.5	3.9
	Rotation ±10°	88.7	**38.6**	**22.3**	**29.2**
	Rotation ±10°, rotation −5°	89.0	36.1	14.2	21.7
	Rotation ±10°, shifting 0.15	87.8	36.8	18.3	26.5
	Rotation ±10°, scaling 0.9	90.2	38.4	10.2	19.7
ConvMixer	Baseline best	91.1	**64.6**	3.6	1.2
	Rotation ±10°	88.5	44.0	**35.9**	**44.8**
	Rotation ±10°, rotation −5°	90.2	38.3	22.4	31.3
	Rotation ±10°, shifting 0.15	86.7	42.9	29.8	38.8
	Rotation ±10°, scaling 0.9	88.4	39.8	28.8	37.1

The bold font represents the highest accuracy.

With more reconstructors, DeR's accuracy under iterative attacks (PGD, AutoAttack) is still higher than baseline methods in most cases. The robustness under the FGSM attack is only slightly lower than that of baseline methods. Although a larger *N* is not better than *N* = 2, the robustness of DeR is still plausible compared to baseline methods, which still verifies the generality of our method.

#### 4.4.6 Reconstructor structure

**Figure 10:**
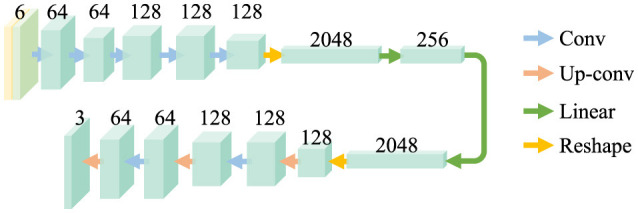
Structure of AutoEncoder.

**Figure 11:**
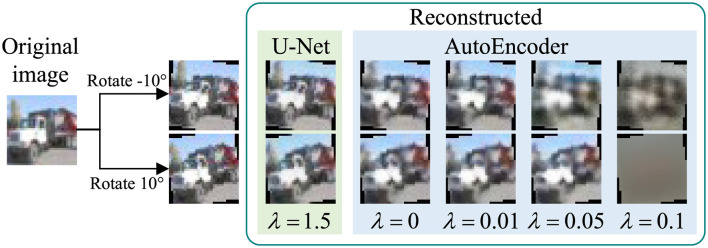
Images reconstructed by U-Net and AutoEncoders (Images reproduced from: Learning Multiple Layers of Features from Tiny Images, Krizhevsky, 2009).

In this section, we implement DeR with another commonly used image reconstruction structure, AutoEncoder. The structure of the AutoEncoder for CIFAR-10 is depicted in Figure 10. The AutoEncoder takes up 7.13 MB of space, larger than the U-Net structure processor, which takes up 2.26 MB. However, the larger parameter volume does not improve reconstruction quality, as illustrated in Figure 11. Although the AutoEncoders are trained with much smaller λ, the fidelity of reconstructed images can not be ensured.

**Figure 12:**
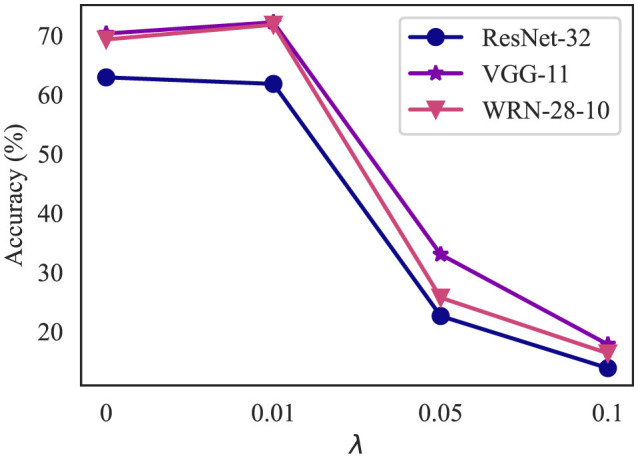
Classifier accuracy on images reconstructed by AutoEncoders.

We further test the classifiers' accuracy on images reconstructed by the AutoEncoders, and the results are shown in Figure 12. The plots show that even when trained with a smaller λ, the AutoEncoders cannot preserve the features of input images. Subsequently, using an AutoEncoder instead of a U-Net to implement DeR defense will harm the classification accuracy. These experimental results indicate that the proposed simplified frequency-aware U-Net structure is more suitable for DeR defense.

## 5 Conclusion

In this study, we propose an efficient plug-in defender, DeR, for adversarial defense. DeR generates diversified gradients by multiple plug-in reconstructors. The reconstructors take a U-Net structure with additional frequency components that generate redundancy for diversifying gradients. By training the reconstructors with DeR loss that combines the reconstruction objective and diversity objective, the reconstructors gain robustness against adversarial attacks while preserving the input fidelity. Extensive experiments under gradient-based attacks on DeR and state-of-the-art plug-in methods demonstrate DeR's superiority in defending adversarial attacks while maintaining input fidelity. The accuracy improvement in AutoAttack under strict settings exceeds 20% on CIFAR-10 and SVHN datasets. Moreover, DeR is simple in model architecture and efficient in inference, which enhances the applicability of DeR in real-time services.

## Data Availability

Publicly available datasets were analyzed in this study. This data can be found at: https://www.cs.toronto.edu/~kriz/cifar.html. The Tiny-ImageNet can be found at: http://cs231n.stanford.edu/tiny-imagenet-200.zip.

## Footnotes

^1^ https://github.com/kentaroy47/vision-transformers-cifar10

^2^ https://github.com/point0bar1/ebm-defense
